# Work Ability and Vitality in Coach Drivers: An RCT to Study the Effectiveness of a Self-Management Intervention during the Peak Season

**DOI:** 10.3390/ijerph16122214

**Published:** 2019-06-22

**Authors:** Art van Schaaijk, Karen Nieuwenhuijsen, Monique Frings-Dresen

**Affiliations:** Amsterdam UMC, University of Amsterdam, Coronel Institute of Occupational Health, Amsterdam Public Health Research Institute, Meibergdreef 9, P.O. Box 22660, 1100 DE Amsterdam, The Netherlands; k.nieuwenhuijsen@amsterdamumc.nl (K.N.); m.frings@amsterdamumc.nl (M.F.-D.)

**Keywords:** e-health, health promotion, prevention, sustainable employment

## Abstract

*Background*: This randomized controlled trial (RCT) evaluates the effectiveness of a self-management toolbox designed to maintain work ability and vitality in coach drivers over their peak season. *Methods*: The intervention group received a self-management intervention providing advice aimed at increasing work ability and vitality. These suggestions targeted three specific domains: work–recovery–rest balance, food and drink intake, and physical activity. At the beginning (March), middle (July), and end (October) of the coach sector peak season, work ability, vitality, work-related fatigue, psychosomatic health, sleep complaints, and perceived mental exertion of coach drivers were assessed through questionnaires. *Results*: A total of 96 drivers participated in the study. Access to the toolbox did not result in significant differences between groups. Work ability and vitality decreased significantly in both groups, falling from 7.8 ± 1.3 to 7.3 ± 1.6 and from 63 ± 16.7 to 55 ± 18.7, respectively. Work-related fatigue increased from 35 ± 31.9 to 52 ± 35.3. Psychosomatic health complaints, sleep complaints, and perceived mental exertion also increased significantly. *Conclusions*: The uptake of the intervention was too low to determine if this toolbox can maintain work ability and vitality in coach drivers when compared with a control group. Overall work ability and vitality decrease significantly as the peak season progresses, while work-related fatigue accumulates. Other interventions should be explored to ensure sustainable employability in this population.

## 1. Introduction

Work ability is of growing importance for individual workers, employers, and sector organizations. The concept of “work ability” is one that is central to research into sustainable employment strategies. The occupational health demands created by a rising pensionable age across many European countries led to growing interest and urgency in this field of study [1]. Work ability describes the capability for satisfactory employee functioning at work while maintaining adequate physical and mental well-being. Sustainable work ability can be threatened if workers struggle or are unable to meet the work demands placed on them because of impaired health, often due to advancing age. Early intervention is, therefore, of critical importance if seeking to optimize work ability; strategies targeting a declining work ability must exert an effect before workers become too incapacitated to function. To prevent the reduction of work ability, preventive efforts should be aimed at an active workforce [2].

Coach drivers are a typical example of a working population at risk of work ability losses. Sustainable good health can be at risk during the annual coach sector peak season, characterized by increased work demands due to long working days starting and ending at irregular hours. During the peak season, the number of rides increases while the number of drivers is limited. According to a study conducted by Schuring et al., the number of working hours per week in the peak season increases by 70% compared to the off-season [3]. During peak seasons, drivers may begin their working day early in the morning one day and late at night the next. The irregular work and sleep patterns that this kind of shift work entails place high demands on the work–recovery–rest balance of drivers [4,5]. This adds to the stress levels already inherent in operating in a road network that is becoming increasingly busy and congested [6]. Additionally, coach drivers must also work within a strict and inflexible schedule when transporting passengers to their destination.

It is, therefore, difficult for drivers to maintain adequate physical and mental health due to the irregular living and eating habits inherent in this working schedule [7,8]. The scope for a regular or healthy eating pattern is small, and this can lead to health complaints [9]. The availability and convenience of unhealthy food on the road makes it challenging for drivers to find healthy food or maintain a healthy diet. Also, due to tight schedules and extended working hours, the window for physical activity is limited. On the road, there may be little time—or opportunity—for leisure activities, and the energy level of a coach driver after a long day is typically too low to be conducive to much physical activity.

The work demands placed on drivers in the coach sector are the same for both younger and older workers; however, the majority of the drivers are over 50 years of age. It is well established that work ability decreases with age; however, in the coach sector, the same work demands have to be met by an aging population of coach drivers [10,11,12].

In addition to reduced load-bearing capabilities, the need for recovery from work is known to be higher in older workers [13,14]. Need for recovery from work was shown to be an indicator of work-related fatigue [15]. Also, importantly, the risk of accidents increases as work-related fatigue increases. It is, therefore, vital to monitor these parameters in persons responsible for the safety of large numbers of people on public roads [16,17]. During the peak season, it can be expected that work ability will decrease when an increase in working hours is ineluctable. However, since work ability is linked to vitality and work-related fatigue [18,19], it can further be predicted that, next to work ability, vitality will drop and work-related fatigue will increase as the peak season progresses and the workload increases [20].

In order to achieve sustainable employability, it is necessary to develop preventive strategies aimed at maintaining work ability and vitality over the peak season. Implementation of such strategies in the coach sector is geographically complicated through drivers being on the road and, as such, it becomes difficult to organize group meetings. Prior research suggested the utility of preventative, tailored interventions that could be applied to each subgroup of drivers [17]. One such tailored strategy, which aims to maintain work ability in coach drivers, is employing the use of a self-management toolbox. Self-management instruments are being more commonly utilized because of technical developments and the increased use of smart devices, allowing them to be used by those without a fixed workplace [21].

An increasingly important field in preventative healthcare strategies is that of “e-health”. Application of this broad discipline to preventative healthcare involves using internet technologies to change behaviors associated with ill health, to deliver preventative healthcare strategies to target populations, and to share healthcare-related information [22]. An example of e-health is development of a specific application as unguided self-management interventions that create awareness of public health messages and distribute information. The goal of unguided self-management interventions is to change behavior and support people without the need for organized meetings or personal contact. These are low-cost interventional methods with the scope to reach large groups of employees. Such methods aim to provide workers with a tool that allows self-optimization of work–life balance and can be conducted at any time and place suitable to the user [23,24,25]. This type of intervention is particularly suited to the working environment of coach drivers and may be able to contribute to behavioral changes facilitating a healthier lifestyle and sustainable work ability [26,27].

It is as yet unknown whether these interventions are useful in maintaining work ability and vitality in coach drivers during their peak season. This led to the formulation of the following research question: Can reductions in coach drivers’ work ability and vitality over the coach sector peak season be prevented through the use of self-management interventions targeting work–recovery–rest balance, eating habits, and physical activity at work?

## 2. Materials and Methods

### 2.1. Participants and Procedure

The study was designed as a randomized controlled trial with equally sized intervention and control arms running in parallel. The follow-up period was seven months (the length of the peak season) and comprised three discrete measurement points. We performed a sample size calculation with a significance level of 0.05 and a power of 0.80. However, the estimated effect size was impossible to predict. When aiming to be able to pick up changes between groups with a medium effect size of 0.5, we estimated needing a group size of 31. Because we did not know what effect size to expect, we chose to include as many drivers as possible for this study to increase the ability of finding differences with a lower effect size. In collaboration with the coach sector organization, we recruited drivers through a variety of strategies—contacting coach companies, visiting events where coach drivers would be present, and placing adverts in job-specific media—over the period from November 2017 to March 2018. After giving informed consent, all drivers who agreed to participate received a baseline questionnaire. No protocol changes were made after the start of the trial.

Baseline questionnaires were administered at the beginning of the peak season (March). The follow-up questionnaires were administered in the middle (July) and at the end of the peak season (October). All questionnaires were digitally administered, meaning that drivers could fill out the questionnaire at the time and location of their choice.

Our research was conducted in accordance with the Declaration of Helsinki [28]. The research proposal was submitted to and approved by the Medical Ethical Committee of the Academic Medical Centre, who decreed that a comprehensive evaluation was not required since this study was not subject to the Medical Research Involving Human Subjects Act (W16_153#16.177). This research was registered in the trial register as NTR 7125. The privacy impact assessment was registered as AMC2017-422. For this study, CONsolidated Standards Of Reporting Trials (CONSORT) checklists for reporting e-health interventions and parallel group randomized trials were used as guidelines [29,30].

### 2.2. Randomisation

All drivers who completed the baseline questionnaire were numbered based on their email addresses. Each number was randomly assigned to either the intervention or control group by an online research randomizer, generating two equally sized lists of numbers (block randomization with blocks of 46 drivers). This was done by an independent researcher (J.S.) who did not possess any personal or other information regarding the drivers nor the allocation details of individual numbers. This researcher reported back the numbers (1–96) as either belonging to the control or intervention group; intervention was subsequently allocated based on these groups.

### 2.3. Measurements

Three questionnaires were developed to study the work-related health of coach drivers. At each of the three time points, work ability, vitality, work-related fatigue, psychosomatic health, sleep complaints, and perceived mental exertion of coach drivers were assessed.

### 2.4. Main Outcome

This trial centers upon the outcome of work ability. Given that the concepts of vitality and work-related fatigue were shown to be closely related to this outcome [18,19], these were also examined. Work ability was measured using the work ability score (WAS) [31], the first question of the work ability index (WAI). In addition to general work ability, physical and mental work ability were also appraised by drivers, using a scale of 0 to 10. These two questions were based on the second question of the WAI, where 0 represents no work ability at all, and 10 describes the best work ability ever experienced [32]. A higher value represents a greater work ability. This single-item assessment showed sufficient convergent validity with the complete WAI and is suitable for the systematic screening of work ability [33].

Vitality was measured with the vitality subscale of the Short Form-36 (SF-36) [34]. Items from this subscale concern user evaluation of levels of energy and fatigue, for example, “How much of the time during the past four weeks did you have a lot of energy?”. This scale comprises four items and gives a total score between 0 and 100. Those with high scores felt lively and energetic over the past four weeks, while those with low scores felt tired and exhausted. The internal consistency of this scale was 0.82 (*α*) and the test–retest correlations were 0.76 and 0.63 after two and six months, respectively [34].

Work-related fatigue was measured through the proxy of need for recovery, using the need for recovery scale of the questionnaire on experience and evaluation of work [15]. This scale contains 11 items and, after transformation, gives a score of between 0 and 100, with a higher score indicating a greater recovery requirement. Need for recovery was shown to be able to predict sickness absence in truck drivers [35]. This scale can be used in both individual and group assessments of need for recovery, with a Cronbach’s *α* value between 0.81 and 0.92 in different subgroups based on education, age, and gender [36].

### 2.5. Secondary Outcomes

In addition to the primary outcomes described above, other factors relevant to work-related health during the coach sector peak season were measured. Psychosomatic health was examined using a questionnaire to measure health complaints (Vragenlijst Onderzoek Ervaren Gezondheid/VOEG) [37,38]. This 13-item dichotomous questionnaire details a number of common (work-related) health complaints (such as fatigue, headache, and back pain). The total sum value is converted to a 0–100 score, with a higher score representing a greater number of health complaints. These 13 items have a Cronbach’s *α* value of 0.67 [37].

Sleep complaints were recorded using the Groningen sleep quality scale (GSKS) [39]. This scale consists of 14 items with a total score of between 0 and 100. The items on this scale are ranked in order of the severity of the complaints, with a greater number of sleep complaints giving a greater score. The Cronbach’s *α* value of this scale is 0.89 [39].

Perceived mental exertion was measured using the perceived mental exertion scale (in Dutch, SEB) [38,40,41]. This scale comprises 19 items in which a driver indicates on a five-point scale which of two answers best describes their situation. From these items, the perceived mental exertion is expressed on a scale between 0 and 100, with a higher score being less favorable. For example, one of the items included in the SEB is “difficulty in planning your own actions vs. working effortlessly”. The Cronbach’s *α* value ranges between 0.95 and 0.97 according to age group and shift work type [41].

### 2.6. Process Measurements

The questionnaire contained questions detailing individual characteristics including age, body mass index (BMI), number of years worked as a coach driver, working hours, sleep hours, work characteristics, regular food and drink intake, and physical activity. This allows for the analysis of driver behavior during the peak season. In addition, changes in behavior can be observed, as well as the points in time that these changes took place. Alongside these characteristics, eight questions were included to examine the influence of work on private life. For example, one of these questions asks “Has your private life been adversely affected by irregular working hours?”.

The follow-up questionnaires distributed to the intervention group also included questions regarding the use of the intervention and its three constituent domains. The process measurements contained questions about the extent to which specific suggestions may have helped preserve vitality the most, and which suggestions were most commonly used.

### 2.7. Intervention

The drivers assigned to the intervention group received a digital self-management toolbox at the start of the peak season after completing the baseline measurements. This toolbox was an interactive pdf document with suggestions and corresponding assignments based around three domains: work–recovery–rest balance, food and drink intake, and physical activity. Drivers were free to print it and write their data and remarks down on paper or use it digitally on any (mobile) device. Each domain of the toolbox included a general introduction of the topic and its importance. After a general introduction, between 11 and 14 (to coach drivers tailored) suggestions were given for each domain, followed by assignments. The advice provided is based on scientific research and is aimed at influencing behavioral changes in order to improve health parameters, and ranging from easy-to-implement tips to suggestions that required more effort, but which could improve health over a longer period. Examples of suggestions include the ideal duration of a power nap [42], ways by which to increase alertness [43], modifying the eating pattern, advice regarding posture, and suggestions for performing quick on-the-spot workouts. Research showed, for example, that physical activity can maintain work ability and reduce levels of work-related fatigue [44]. Each of the three domains of the toolbox was preceded by suggestions for intrinsic motivation, and drivers were asked to record their motivations for engaging with the toolbox. The suggestions provided in the toolbox were derived through consulting professionals in the relevant scientific fields, such as a dieticians and movement scientists. In the assignments, drivers were required to record their behaviors, set a goal, and indicate which suggestion they intended to use and why they felt that this was going to be successful. In addition, the toolkit sought to include the concept of “peer support”. Drivers were divided into groups of three, all working over the peak season, and were instructed to keep in regular contact through the assignments in order to motivate each other, to encourage engagement with the toolbox, and for evaluative purposes. Peer support and interpersonal contact were previously shown to be of benefit in stimulating behavioral changes in e-health interventions [22]. There was no contact with researchers or other professionals after the distribution of the toolbox to the intervention group, other than through the digital questionnaires to evaluate the intervention.

### 2.8. Statistical Analysis

In order to analyze the differences between the intervention and control groups over the measurement period, analyses of covariance (ANCOVA) were used. Differences in baseline measurements were corrected for using the baseline values as covariates, with the grouping variable as the fixed factor. The assumptions of the ANCOVA analyses were tested to make sure no abnormalities in the data were observed [45]. For the analysis, an intention-to-treat analysis was used to study the results as measured.

To identify temporal trends in work ability, vitality, and work-related fatigue during the peak season, data from the baseline, intermediate, and final measurements were compared using different paired sample *t*-tests. This allowed the identification of changes within groups that occur between the three time points during the peak season. Differences in recovery opportunities between the three measurements were tested with McNemar tests for binary paired outcomes.

To check for selective dropout, a missing values analysis was performed. To control for missing values, an additional analysis was performed with the last observation carried forward method to correct for missing data in a conservative way, when representing data of the entire population.

### 2.9. Secondary Analyses

A separate secondary analysis was performed to check for the influence of compliance with the intervention on the outcome measures. In the per-protocol analysis, only the participants who complied with the protocol were considered (sufficient use of the toolbox: >10%).

### 2.10. Process Evaluation

In an additional process evaluation, we asked whether or not the drivers had the feeling that the tips from the toolbox helped them to feel more vital. This was done for each individual tip per domain, where drivers could indicate what domain they used the most, and which tips within the domain were most helpful.

## 3. Results

A total of 124 of an estimated eligible 6000 drivers working in the private passenger transport sector gave informed consent to participate in this study. The exact number of drivers invited is unknown because the coach sector organization was responsible for inclusion of drivers due to privacy data sharing restrictions. These drivers received the first questionnaire in March 2018. The 96 drivers who completed the baseline questionnaire were randomly assigned to either the intervention or control group, creating two equal groups of 48 drivers. Of the 96 drivers, the average age was 53 ± 10.8 years, and 85% were male. Mean BMI was 28 ± 4.6, and drivers were working as a coach driver for an average of 16 ± 11.2 years. In total, 28% drove shuttle services and multi-day trips, 24% drove single-day trips, and 48% drove a combination of both. Population demographics are shown in Table 1.

**Figure 1:**
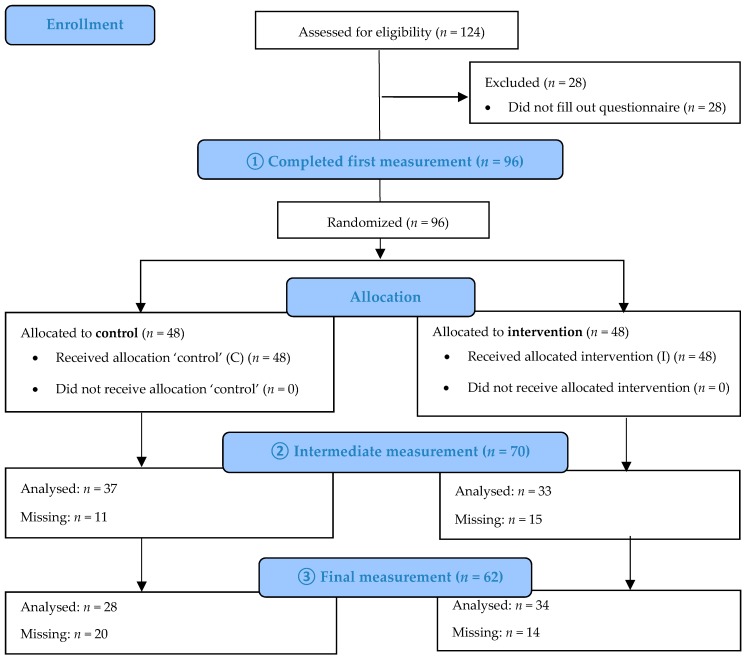
Flow diagram of the enrollment, allocation, follow-up, and analysis process.

At the second time point, in the middle of the peak season (July 2018), 70 drivers (33 from the intervention arm; 37 from the control arm) filled out the questionnaire. A total of 62 drivers (34 from the intervention arm; 28 from the control arm) completed the final questionnaire at the end of the peak season (October 2018). A flow diagram of driver participation is shown in Figure 1.

### 3.1. Differences between Intervention and Control Groups

At baseline, no significant differences between the control and intervention group were observed for any of the variables. Both primary and secondary outcomes of drivers who received the intervention did not differ from the control group over the measurement period (in either the intermediate or final measurements). Access to the toolbox was not associated with any significant change in measures of work ability, vitality, work-related fatigue, psychosomatic health, sleep complaints, or perceived mental exertion, recorded at the middle and end of the peak season when corrected for the baseline score (Table 2). The effect sizes ranged between 0 and 3.5%, demonstrating that use of the toolbox had no effect on the primary and secondary outcomes.

No significant differences between intervention and control groups were found in any of the parameters at any of the three measurement points. Food and drink intake did not change significantly between the intervention and control group. The number of drivers not being physically active increased significantly over the measurement period in both groups, as did the number of hours worked. Neither of these trends, however, differed significantly between groups. The mean number of hours of sleep per night remained unchanged over the measurement periods in both groups. These values are shown in Table 3. In a secondary analysis using the per protocol analyses, no significant differences in the primary and secondary outcomes were found between the intervention and control groups. The numbers of drivers included in the per protocol analysis were 26 and 47 for the intermediate measurement, and 26 and 36 in the final measurement for the intervention and control group, respectively.

### 3.2. Trajectory of Work Ability, Vitality, and Work-Related Fatigue

**Figure 2:**
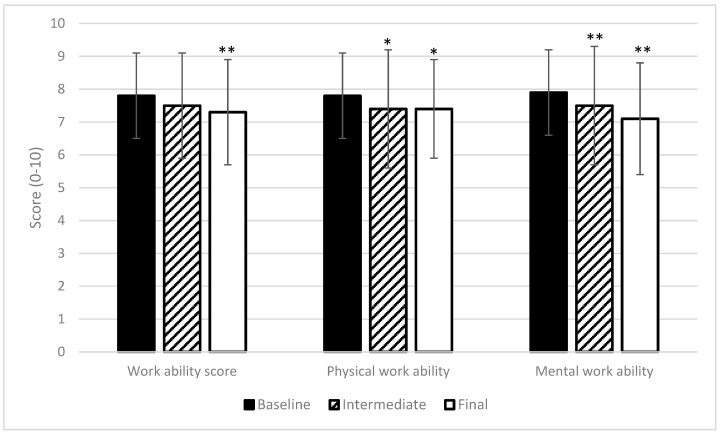
Trajectory of general, physical, and mental work ability during the peak season. Mean and SD are shown with error bars. A score of 0 stands for no work ability at all, and 10 corresponds to the best work ability ever experienced. Values with * or ** showed significant changes compared to baseline measurements: * *p* < 0.05, ** *p* < 0.01.

**Figure 3:**
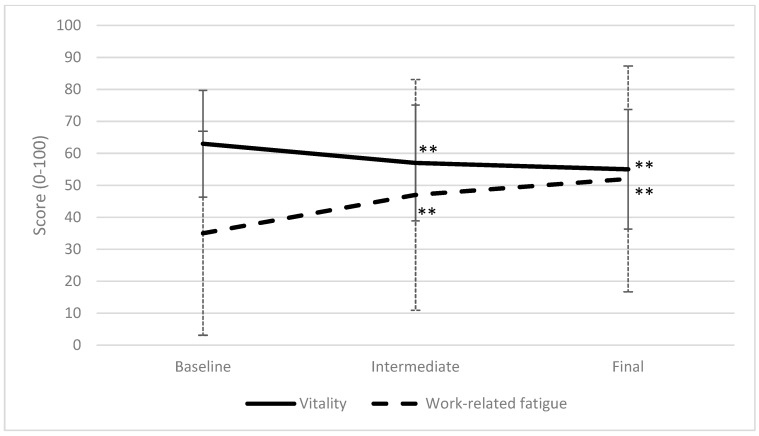
Trajectory of vitality and work-related fatigue during the peak season. Mean and SD are shown with error bars. A lower score indicates a lower vitality and less work-related fatigue. Values with ** showed significant changes compared to baseline measurements: ** *p* < 0.01.

Because there were no significant changes between the intervention and control group over the measurement period, the combined cohort of coach drivers was evaluated as one group. For this group, a decline in work ability and vitality, and an increase in work-related fatigue were evident. Figure 2 and Figure 3 illustrate these changes in primary outcomes. Also, the secondary outcomes recorded also demonstrate a global decline in health parameters. Psychosomatic health complaints increased from 26 (at baseline measurement) to 38, on a scale of 0 to 100 (*p* < 0.01). Sleep complaints and perceived mental exertion increased from 22 to 31, and from 26 to 31, respectively (*p* < 0.05).

The work ability score decreased significantly from 7.8 at baseline to 7.3 at the final measurement of the peak season. It is noteworthy that mental work ability declined the most for all work ability parameters recorded, falling from 7.9 at baseline to 7.1 at the final measurement, while physical work ability was reduced from 7.8 to 7.4.

Vitality decreased, and work-related fatigue increased most dramatically over the period preceding the intermediate measurement, with vitality falling from 63 to 55 and fatigue increasing from 35 to 47 between baseline and intermediate measurements. The values of the final measurements were all significantly different from the baseline measurements. This is depicted in Figure 3.

The values in Figure 2 and Figure 3 illustrate that all values changed in the adverse direction over the measurement period. Aside from the primary and secondary outcomes, recovery opportunities also became limited during the peak season. This shows that there is less time for leisure due to increased working hours while maintaining the hours of sleep (Table 3). Driver perception of recovery opportunities is shown in Table 4. Statistical differences were calculated only between drivers that filled out both questionnaires.

Table 4 illustrates that drivers perceived the number of recovery opportunities to decrease as the peak season progressed. Table 4 presents percentages derived from the entire driver population participating at that time point. For statistical analyses, only the drivers that completed both questionnaires were included, to allow a true comparison of changes in driver perception (i.e., comparing the same divers at both measurement points) to eliminate bias.

On average, drivers in the intervention group reported that they used 34 ± 25.5% and 34 ± 20.0% of the suggestions included in the toolbox during the periods preceding the intermediate and final measurements (Table 5). The process evaluation showed that the domain and corresponding tips on food and drink uptake were used most. The tips that were most helpful for each domain were keeping contact with passengers and colleagues to remain alert on the road in the work–recovery–rest domain, suggestions to have variations in food and drink intake in the food and drink intake domain, and a tip to improve posture during driving and other work activities in the physical activity domain.

Although the mean usage of the toolbox was not very high, drivers reported an increase in vitality attained by applying the advice given in the toolbox. Most of the drivers did not engage in peer support, but those who did (18%) contacted their peers more than four times per month.

The driver-perceived effectiveness of the toolbox on vitality is presented in Table 6. Despite the low usage, some drivers described feelings of maintained or increased vitality. Only one driver reported a reduction in perceived vitality following the use of the toolbox.

## 4. Discussion

### 4.1. Key Results

A specifically designed toolbox based on principles of self-management did not lead to maintained levels of work ability, vitality, work-related fatigue, psychosomatic health, sleep complaints, or perceived mental exertion during the peak season. All measured work-related aspects of health showed significant deterioration as the peak season progressed, regardless of whether the driver received the toolbox. The process evaluation revealed that coach drivers used the toolbox to such a limited extent that differences in these parameters between intervention and control groups were unlikely to occur.

### 4.2. Comparison with Literature

Compared to a study conducted during the (August) 1996 coach sector peak season [46], greater values were obtained for work-related fatigue in this study of the 2018 peak season. Work-related fatigue was 33 at the end of the peak season of 1996, measured on the same scale as used in our research, while the score at the start of the peak season of 2018 was 35, increasing to 52 over the measurement period. Psychosomatic health, sleep complaints, and experienced mental stress at the end of the peak season were also higher than the values obtained in 1996 (24, 22, and 27, respectively). It is hard to pinpoint the causes for these differences, but they may be caused by an increased workload and reduced driver capabilities for dealing with this due to more advanced age. The average age of participating drivers increased by nine years from the 1996 study (53 compared to 44 years of age).

With an average WAS of 7.3 at the end of the peak season, the work ability values of this study are considered moderate in the classification of Gould et al. (2008) [47]. A mean WAS score of 7.3 is considerably lower than scores for ambulance workers, an average working population, a heterogeneous sample of workers, elderly construction workers, or elderly workers, (8.5, 8.1, 7.95, 8.0, and 8.57, respectively). The score is comparable to people returning to work after sick leave (7.4) [32,33,48,49,50,51]. Vitality scores at the beginning of the peak season are comparable with values found in the literature [52]. These values in the literature are averages at a certain point and do not reflect a period with increased workload.

### 4.3. Strengths and Limitations

A strength of this study was that it adhered strictly to the recommendations for randomized controlled trials (RCTs). For instance, randomization took place after the baseline measurement, eliminating any selection bias [53]. Individual differences in baseline values were controlled for by using the baseline values as covariates to avoid conditional bias. To enhance the quality of the intervention, recommendations for self-management interventions were implemented in a structured way. In self-management interventions, it is important to have a strong theoretical foundation, carefully designed structure, and pathway of action, and to include user reflection on behavior [21]. The toolbox aimed to increase intrinsic motivation and reflection on current and desired driver behavior. Previous research studied measures that can promote health and work ability in truck drivers [54]. The authors considered several criteria to be of importance in this study: a wide range of options, measures that help overcome obstructions, and the inclusion of educational material. The final criterion is that both employer and employee are involved. The toolbox presented in our study met all criteria other than this final one. The reason to not actively involve the employer was that it was the wish of the coach sector organization to implement an intervention that drivers could use on their work ability without the assistance of other parties.

A positive attribute of this study was that it succeeded in conducting an RCT in a population with a high workload, minimal free time, irregular hours, and no fixed workplace location. This study stayed close to practice while following the methodological design. A realistic intervention was implemented in such a way that allowed the effects of this intervention to be monitored over the peak season. High external validity was established because there was no check on compliance, as there would not be any checks after this toolbox is implemented in the coach sector in the future; therefore, the true effects of this intervention were studied. It is important to keep in mind that the intention was for the intervention to work under the conditions of normal practice and not in a restricted controlled situation.

This study aimed to include as many coach drivers working over the peak season as possible. However, the sample size was still low. One reason for this may be the difficulty of reaching these coach drivers. The coach sector association only had contact details for individual coach companies, and not of individual drivers. The low sample size did not affect the results because no trend was observed in the differences in primary or secondary outcomes between intervention and control groups. The small effect size predicts that even a larger sample size would not lead to significant results. Despite the small sample size, significant trends can be seen when examining the entire cohort of drivers over time. In our measurement process, we chose to include all drivers that filled out questionnaires instead of only the drivers that filled out all questionnaires in order to avoid bias; not doing so would have allowed selective dropout (i.e., sick leave) to change the mean values at baseline. The intention-to-treat analysis showed the population as it was during the peak season.

### 4.4. Generalizability

We would expect the outcomes of this study to apply to the entire population of Dutch coach drivers, as participating drivers were drawn from a number of different companies and locations. The high average age and percentage of males in the cohort are representative of the wider Dutch coach driver population. Selection bias was eliminated through randomization to intervention and control groups. It is plausible that some drivers did not participate in the study because they felt too busy during the peak season, or that only drivers who were busy during the peak season participated because they endorsed the importance of this study.

Unfortunately, we do not have information regarding the reasons for dropout. There was no attrition bias between groups; however, when selective dropout rates were examined, those who dropped out after the baseline measurement possessed more positive baseline values. This would indicate that the worsening in health parameters seen over the study period is slightly exaggerated. However, additional conservative analyses with the ‘last observation carried forward’-method still showed significant changes in the negative direction on both the primary and secondary outcomes (see Table A1). These values are without a possible decrease in health parameters in the higher values of the drivers that discontinued after baseline measurement. The values analyzed using the last observation carried forward method are, therefore, an underestimation of the effect of working during the peak season on health parameters. In their potential impact on value differences between groups, we would expect this effect to be minor and balance out between groups because of randomization.

The results of this study can be extrapolated to other countries within the European Union, as the driving and rest times for drivers are controlled by European legislation (EG nr. 561/2006) [55]. We would expect that the working conditions of drivers in other European countries are comparable and drivers experience similar difficulties. In the United States, Canada, and Australia, however, the maximum number of driving hours per day permitted is higher than in Europe [56,57,58]. Therefore, it can be hypothesized that, if these driving hours are also irregular, sustainable employment may be harder to achieve in drivers from these countries.

In The Netherlands, the number of rest hours required between journeys was increased over 20 years ago through a collective labor agreement. A study found that drivers who received more rest time between trips had lesser values for work-related fatigue and fewer psychosomatic health complaints [3]. Since this change in legislation, driver workload may have increased, resulting in an increased need for recovery.

In other driving occupations, the same rules apply that govern working hours. However, work ability and work-related fatigue in these professions may benefit from more predictable start and end times, or a more consistent schedule. Coach drivers also have to take a large number of passengers and their wishes into account, which may cause extra stress.

### 4.5. Interpretation

The results of this study clearly demonstrate a significant downward trend in health parameters during the peak season. Values at the start of the peak season were comparable to other working populations as stated above. However, toward the end of the peak season, these values became alarming. The percentage of individuals exhibiting poor work ability at the start of the peak season was 6%. This increased to 11% at the end of the peak season. Although this increase may seem low, this represents nearly a twofold increase of workers at risk of sick leave. Our findings show that 20 to 27% of drivers had a relevant decrease in work ability (general, mental, or physical) over the peak season [49]. Although the mean work ability score was still acceptable, 35% of drivers scored higher than the threshold (over 50) for work-related fatigue at baseline. This percentage increased to 52% at the intermediate measurement and 61% at the final measurement, illustrating that the majority of drivers experience too much work-related fatigue. Since work-related fatigue is related to occupational accidents, these figures underline the necessity for interventions in the coach sector [16,17,59]. Vitality decreased over time; however, a cutoff point was not determined. Since significant decreases occurred in vitality between measurements, this should be interpreted as significantly more fatigue corresponding to the baseline scores in Table 2.

Personal demographic characteristics might explain work-related fatigue, as it is known that older workers have a higher need for recovery [13]. Also, BMI can cause difficulties in certain parts of work as a coach driver. Since gender and BMI did not change over the peak season and the analyses were corrected for baseline values, the changes in the primary and secondary variables were not attributable to these characteristics. In an additional analysis, we found no evidence for influence of BMI, gender, and age on the decrease in work ability, vitality, and increase in work-related fatigue. Future studies may shift focus toward which personal and work aspects cause the work ability and vitality to decrease and the work-related fatigue to increase.

The fact that the work-related health of a large proportion of drivers can worsen significantly over a relatively short period demonstrates that measures to improve sustainable employability are of great importance in this sector.

### 4.6. Implications

Although no significant differences were found between the intervention and control group, our findings show the need for interventions to combat reductions in work ability during the peak season. Such interventions should be aimed at improving sustainable employment in an aging workforce of coach drivers. Given the increased average age in this population (two-thirds are now older than 50 years, half of whom are also older than 60 years), ensuring sustainable employability is of particular importance because the load-bearing capacity decreases on average. This, in combination with the increased work-related fatigue and a higher mental workload experienced by older drivers, has the potential to result in driving errors that may endanger the safety of both passengers and other road users [8,60,61].

Previous research advised that monitoring work-related fatigue can help employers and occupational health agencies to develop preventative strategies to increase sustainable employability, given that the need for recovery is an important predictor of future sickness absence [35,62]. A study in older taxi drivers recommended preventive screening and early interventions [63]. This screening can be in the form of a preventive medical examination with an associated advice on identified problem areas. Since the reductions in the mean health parameter scores observed in this research were not only attributable to a few low-scoring drivers, interventions should be aimed at the entire coach driver workforce. Future research should aim to find alternate ways to maintain work ability and to improve sustainable employability since the use of a self-management intervention did not yield the desired results. The effectiveness of a more active approach should be tested in the future.

Work ability score, vitality, work-related fatigue, psychosomatic health, sleep complaints, and perceived mental exertion were proven to be useful outcome measures, which can be used in future research because they are able to detect changes over the peak season. Future effectiveness studies for this group should, therefore, include these measurements during the peak season in order to demonstrate the preventive effect of interventions.

Research on the effective underlying mechanisms of interventions is desired in a population that is always on the road in order to improve the preventive effect of interventions. Additional research on the personal characteristics that influence the main outcomes can contribute to improving occupational care and improving sustainable employability. In research in this older population, it is important to address the aspects that may affect the effectiveness of interventions. Therefore, it is suggested to shift the focus of research toward identifying the working component in interventions while still focusing on health improvement.

## 5. Conclusions

A decrease in work ability was not prevented by means of a self-management toolbox in this study. Uptake of the intervention was too low to be able to reliably determine whether such an intervention could lead to an effect between groups. User evaluation showed that the content of the intervention was considered to be positive, but without yielding any significant results. Overall work ability and vitality decreased significantly, and work-related fatigue accumulated as the peak season progressed. Passive intervention with the interactive toolbox was not used enough in this study population and, therefore, the coach sector should explore active interventions to ensure that work ability is maintained in the peak season and that long-term sustainable employability is attained.

## Figures and Tables

**Table 1:** Population demographics (mean and standard deviation) for the intervention and control groups.

Demographic	Intervention Group	Control Group
Number of drivers	48	48
Percentage of male drivers	88	83
Age	55 (9.1)	52 (12.1)
Body mass index (BMI)	28 (4.6)	28 (4.6)
Years working as coach driver	14 (11.1)	17 (11.2)
Percentage not physically active at baseline	31	31

**Table 2:** Values (mean ± SD) for the primary and secondary outcome variables for the intervention and control group at the baseline, intermediate, and final measurement, based on the intention-to-treat protocol. The estimated effect size, significance (*p*-value), and F-value of the ANCOVA analyses of both analyses are shown in the right columns. Both the intention-to-treat and the per protocol analysis results are presented.

Outcome Measure	Baseline		Intermediate		Final		Est. Effect Size		Sign.		F	
	Int.	Contr.	Int.	Contr.	Int.	Contr.	ITT	PP	ITT	PP	ITT	PP
Number of drivers	48	48	33	37	28	34						
Work ability score	7.8 (1.4)	7.8 (1.2)	7.7 (1.5)	7.3 (1.6)	7.0 (1.5)	7.5 (1.6)	0.021	0.016	0.261	0.328	1.290	0.972
Physical work ability	7.8 (1.3)	7.8 (1.3)	7.5 (1.9)	7.2 (1.7)	7.3 (1.6)	7.5 (1.4)	0.000	0.000	0.945	0.894	0.005	0.018
Mental work ability	7.9 (1.2)	7.8 (1.4)	7.5 (1.8)	7.4 (1.8)	6.9 (1.8)	7.2 (1.5)	0.009	0.012	0.473	0.393	0.522	0.742
Vitality	63 (15.2)	63 (18.2)	57 (18.4)	57 (18.1)	55 (20.1)	54 (17.8)	0.000	0.010	0.954	0.934	0.003	0.007
Work-related fatigue	33 (30.6)	37 (33.0)	46 (35.6)	48 (37.0)	53 (34.5)	52 (36.6)	0.002	0.010	0.719	0.447	0.131	0.587
Psychosomatic health	25 (19.7)	28 (22.0)	30 (24.6)	31 (24.0)	36 (27.1)	39 (25.4)	0.001	0.001	0.806	0.773	0.806	0.084
Sleep complaints	19 (23.3)	26 (24.6)	23 (21.3)	36 (29.8)	28 (25.8)	33 (29.7)	0.001	0.000	0.788	0.903	0.073	0.015
Perceived mental exertion	25 (19.2)	26 (19.6)	28 (20.9)	32 (22.1)	27 (16.5)	33 (23.5)	0.035	0.031	0.151	0.176	2.121	1.878

Int. = intervention group, Contr. = control group, ITT = intention-to-treat analysis, PP = per protocol analysis. Estimated effect size shown as partial eta squared, Sign. = significance level, F = F-statistic of ANCOVA.

**Table 3:** Values (mean ± SD or %) for the process measures for the entire group of drivers at the baseline, intermediate, and final measurements.

Process Measure	Baseline	Intermediate	Final
Number of drivers	96	70	62
Work hours per week ^1^	37 (15.4)	50 (18.4) **	45 (17.6) **
Hours of sleep ^2^	7.8 (2.2)	7.7 (2.9)	7.8 (2.6)
Not physically active (%) ^3^	31	42	52 *
Trouble staying alert during evening and night hours (%) ^3^	29	36	42
Self-assessed as very tired (%) ^3^	22	41 *	53 **

^1^ Mean of last three weeks, ^2^ between last two working days, ^3^ during the last two weeks. Significant differences compared to baseline measurement are marked with asterisks: * *p* < 0.05, ** *p* < 0.01. For the McNemar test, baseline–intermediate *n* = 70, baseline–final *n* = 62.

**Table 4:** Percentage of drivers that answered “yes” on the corresponding questions on recovery opportunities.

Recovery Opportunity	Baseline	Intermediate	Final
Number of drivers	96	68	62
Could you interrupt your work at times when you felt it necessary?	55	46	50
Could you determine the start and end time of your work yourself?	11	12	10
Could you decide when you took a break?	38	44	45
Could you include a separate day off when you wanted?	60	38 **	44
Have you been recalled from leave/a free day?	13	18 *	16
Were your work and rest times well organized?	91	82	76
Were there opportunities for you to work at hours that fit your private situation?	69	43 **	42 **
Has your private life been adversely affected by irregular working hours?	45	53	61

Significant differences compared to baseline measurement are marked with asterisks: * *p* < 0.05, ** *p* < 0.01. For the McNemar test, baseline–intermediate *n* = 68, baseline–final *n* = 62.

**Table 5:** Usage (mean % ± SD) of the separate domains in the toolbox in the intervention group in the intermediate and final measurements.

Toolbox Domain	Intermediate	Final
Number of drivers	30	28
Work–recovery–rest balance	32 (27.6)	31 (22.3)
Food and drink intake	38 (32.0)	38 (27.8)
Physical activity	31 (24.7)	32 (20.4)

**Table 6:** Perception of effectiveness (*N* (%)) of the toolbox on vitality according to the drivers’ perception in the intermediate and final measurements, based on the following question: Do you have the feeling that you are more vital now then you would have been without the use of the toolbox?

Perceived Vitality	Intermediate	Final
Number of drivers	30	28
Less vital	0 (0)	1 (4)
Equally vital	17 (55)	15 (54)
More vital	4 (14)	3 (11)
I do not know	9 (31)	9 (32)

## Appendix A

**Table A1:** Values (mean ± SD) for the outcome variables for the entire group of drivers at the baseline, intermediate, and final measurements, with the last observation carried forward and-intention to-treat analysis.

Outcome Measure	Baseline	Intermediate	Final
Number of drivers	96	96	96
Work ability score	7.8 (1.3)	7.6 (1.4)	7.5 (1.4) *
Physical work ability	7.8 (1.3)	7.5 (1.7) *	7.5 (1.5) *
Mental work ability	7.9 (1.3)	7.6 (1.6) **	7.3 (1.6) **
Vitality	63 (16.7)	58 (17.1) **	58 (17.4) **
Work-related fatigue	35 (31.9)	44 (34.5) **	45 (34.2) **
Psychosomatic health	26 (20.8)	28 (23.1)	31 (24.6) **
Sleep complaints	22 (24.0)	27 (25.2) *	27 (25.6) *
Perceived mental exertion	26 (19.3)	29 (21.3)	30 (21.4) *

Values with * or ** showed significant changes compared to baseline measurements: * *p* < 0.05, ** *p* < 0.01.
